# Protein, Amino Acid, Fatty Acid Composition, and in Vitro Digestibility of Bread Fortified with *Oncorhynchus tschawytscha* Powder

**DOI:** 10.3390/nu10121923

**Published:** 2018-12-05

**Authors:** Ajay S. Desai, Tang Beibeia, Margaret A. Brennan, Xinbo Guo, Xin-An Zeng, Charles S. Brennan

**Affiliations:** 1 School of Food Science and Engineering, South China University of Technology, Guangzhou 510640, China; ajay.desai@lincolnuni.ac.nz (A.S.D.); margaret.brennan@lincoln.ac.nz (M.A.B.); guoxinbo@scut.edu.cn (X.G.); xazeng@scut.edu.cn (X.-A.Z.); 2 Department of Wine, Food and Molecular Biosciences, Lincoln University, Christchurch 7647, New Zealand; beibei.tang@lincolnuni.ac.nz; 3 Riddet Research Institute, Palmerston North 4442, New Zealand

**Keywords:** bread, salmon powder, protein, fatty acids, in vitro starch digestibility, in vitro protein digestibility, antioxidant activity

## Abstract

This study investigated protein, amino acid, fatty acid composition, in vitro starch and protein digestibility, and phenolic and antioxidant composition of bread fortified with salmon fish (*Oncorhynchus tschawytscha*) powder (SFP). The proximate composition in control and SFP breads ranged between (34.00 ± 0.55–31.42 ± 0.73%) moisture, (13.91 ± 0.19–20.04 ± 0.10%) protein, (3.86 ± 0.02–9.13 ± 0.02%) fat, (2.13 ± 0.02–2.42 ± 0.09%) ash, (80.10 ± 0.018–68.42 ± 0.11%) carbohydrate, and (410.8 ± 0.18–435.96 ± 0.36 kcal) energy. The essential amino acids of the control and SFP breads ranged between 261.75 ± 9.23 and 306.96 ± 6.76 mg/g protein, which satisfies the score recommended by FAO/WHO/UNU (2007). Protein digestibility of the products was assessed using an in vitro assay. The protein digestibility, comma, amino acid score, essential amino acid index, biological value, and nutritional index ranged between 79.96 ± 0.65–80.80 ± 0.99%, 0.15 ± 0.06–0.42 ± 0.06%, 62.51 ± 1.15–76.68 ± 1.40%, 56.44 ± 1.05–71.68 ± 1.10%, 8.69 ± 0.10–15.36 ± 0.21%, respectively. Control and SFP breads contained 60.31 ± 0.21–43.60 ± 0.35 g/100 g total fatty acids (saturated fatty acids) and 13.51 ± 0.10–17.00 ± 0.09 g/100 g total fatty acids (polyunsaturated fatty acids), and SFP breads fulfil the ω-6/ω-3 score recommended by food authority. There was a significant effect of SFP on bread-specific volume, crumb color, and textural properties. The in vitro starch digestibility results illustrate that the incorporation of SFP into wheat bread decreased the potential glycemic response of bread and increased the antioxidant capacity of bread. In conclusion, this nutrient-rich SFP bread has the potential to be a technological alternative for the food industry.

## 1. Introduction

The high protein values of fish make it an important dietary source of essential amino acids (lysine, methionine, and threonine), as well as a good source of ω-3 fatty acids, especially eicosapentaenoic acid (EPA) and docosahexaenoic acid (DHA). These nutrients have been shown to have a positive effect on human health, such as prevention of cardiovascular disease, hypertension, cancer, and diabetes, as well as containing micronutrients such as vitamins (A, D, B6, and B12) and minerals (iron, zinc, iodine, selenium, potassium, and sodium) [1]. The American Heart Association (AHA) has suggested that in order to achieve benefits for the protection of the heart, consumers should eat at least two servings of fish per week (200 mg/day of long chain ω-3 polyunsaturated fatty acid-PUFA). The human body is unable to synthesize EPA and DHA, and therefore these nutrients need to be acquired through dietary interventions. One such dietary intervention could be the fortification of common food products with fish powder. The high protein content of fish powder may also have an effect in reducing the glycemic index of foods and this in turn may have a potential beneficial health effect with regard to weight control and obesity [2]. Research has shown that ω-3-enriched fish powder can reduce the oxidation of lipids while exposed to high temperatures, such as during baking [3]. Fish proteins also exhibit antioxidant activities, which may be used to control health diseases via the reduction of oxidative stress [4]. Data suggests that in Western diets, there are higher amounts of ω-6 than ω-3 fatty acids, and that the combination of these fatty acids are not well balanced. Throughout the world, bread is an important staple food and widely consumed, and are made from grain-based carbohydrates [5]. These cereal products are often low in protein, vitamins, and minerals, and are usually deficient in essential amino acids and fatty acids and, therefore, are not a balanced food [6]. Due to their relatively low cost, availability, acceptability, and widespread consumption, cereal food products are considered to be one the best vehicles for food fortification [7]. It is possible to improve consumer’s health by fortifying these products with biological ingredients; however, such fortification can have effects on the physical properties of foods, such as the structure of the dough matrix, which in turn affects the physical and nutritional properties of wheat bread by disturbing the viscoelastic network through the dilution of the gluten structure [8]. Proteins and amino acid derived from fish are considered nutritionally superior to that of plant origin ingredients. As consumer attention has become focused on the prevention of diseases, such as cardiovascular disease, type-2 diabetes, colon cancer, and obesity through diet [9], there has been an increased demand for enrichment of food with improved physical (volume, color, and texture), nutritional (amino acid and fatty acid content, protein digestibility, and starch digestibility), and antioxidant properties [10]. Low glycemic index foods can be achieved through the utilization of protein and lipid rich ingredients combined with cereal gains in products such as bread. Phenolic compounds exhibit biological properties, such as antioxidant activity. Food rich in polyphenols have the potential to protect against various diseases associated with oxidative damage, such as cancer, and cardiovascular and neurological disease [11]. In recent years, to achieve this, many nutritional ingredients such as carob [12], flaxseed and lupin [5], mushroom powder [6], cobia [13], and *Chlorella vulgaris* [8] have been incorporated into bread to improve its nutritional composition and product quality. Other products, such as pasta [14] and pizza [15], have been developed with the inclusion of fish powder in their formulation. However, the technological and nutritional properties of bread fortified with partial replacement of wheat flour by salmon fish (*O. tschawytscha*) powder (SFP) are still unknown. Therefore, this study evaluated the effects of fortification of different levels SFP on the bread nutritional quality including protein, amino acid, fatty acid composition, and in vitro starch and protein digestibility, i.e., the physical characteristics, and its technological and antioxidant properties of bread.

## 2. Materials and Methods

### 2.1. Raw Materials

All the ingredients were obtained from a local supermarket (Foodstuffs, Christchurch, New Zealand) including wheat flour (Champion Flour Milling Ltd., Christchurch, New Zealand), sugar, yeast powder, butter, and salt. The salmon fish material (*O. tschawytscha*) was bought from Akaroa Salmon Ltd., (Christchurch, New Zealand).

### 2.2. Fish Powder Preparation

**Figure 1:**
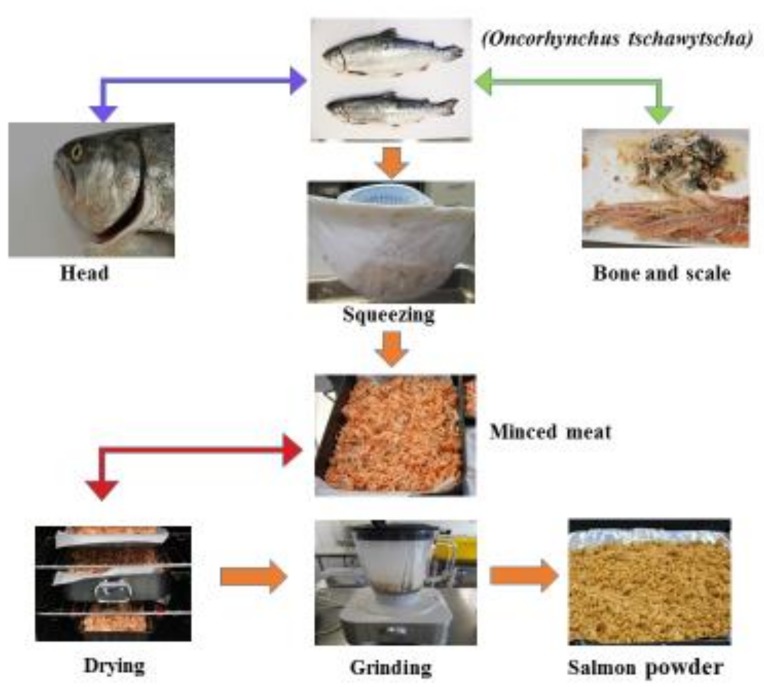
Salmon fish powder.

Salmon meat was prepared from the meat components of the fish after deheading and deboning. The material was cooked in boiling water for 5 min, dried in a temperature-controlled cabinet for 40 h at 50 °C (Moffat, E32M, Christchurch, New Zealand) and milled into a fine powder (mesh: 500 micron) to obtain the salmon fish powder (SFP) [16]. The powder (Figure 1) was stored at (−20 °C) until required.

### 2.3. Preparation of Bread

**Figure 2:**
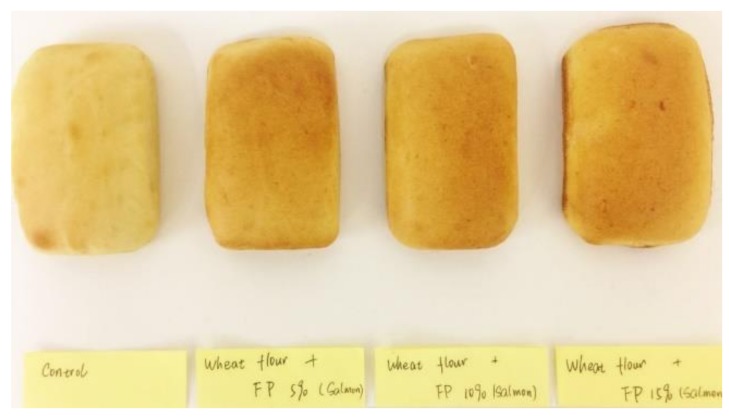
Bread fortified with SFP.

The formulation for the bread products is shown in Table 1A and is derived from a previous study [17]. Wheat flour was replaced by varying levels of SFP in the dough (Figure 2) at concentrations of 0, 5, 10, and 15% *w/w* based on wheat flour dry weight. The dough was formed by using a mixer (Delta Planetary Mixer (ED5), Southern Hospitality Ltd., Christchurch, New Zealand) to mix for 10 min. It was then kneaded by hand for 5 min and rested at room temperature for 5 min. The dough was molded into small loaves (50 g each) and proved at 35 °C for 45 min (Manitowoc, OEB 6.10, Wolfratshausen, Germany), rested in the prover for 15 min, and then baked at 180 °C in an electric oven (Model: E32M, Moffat Ltd., Christchurch, New Zealand) for 15 min. The bread samples were allowed to cool for 2 h after baking.

### 2.4. Proximate Chemical Composition Analysis of Bread

The AACC method 976.05 (2000) was used to determine the protein content using a protein conversion factor of 6.25. The Soxhlet extraction method determined the fat content of the samples, and ash content of the materials was determined according to our previously published methods [18]. The carbohydrate content was estimated by subtracting the total fat content, protein content, ash, and moisture content from 100%. The energy valve was calculated using the formula described in Reference [18]:

Energy value (kcal/100 g) = 4 × protein (%) + 9 × lipid (%) + 4 × carbohydrate (%)(1)

### 2.5. Moisture Content

The moisture content of the bread was determined using the oven-drying method, and the general operation procedures for this method described in Reference [19]:

(2) $$\mathrm{Moisture}\;(\%)\;=\;\frac{weight\;of\;fresh\;sample\;-\;weight\;of\;dried\;sample}{weight\;of\;sample}\times \;100$$

### 2.6. Volume, Density, and Texture Properties of Bread

Bread height, specific volume, texture, and color attributes were all measured. The bread volume was determined by the rapeseed displacement method, following the (AACC, 2000). The specific volume of bread was obtained through dividing the bread volume (mL) by the bread weight (g). Texture analysis of the bread was determined on slices of 25 mm thickness, using a texture analyzer (TA.XT2, Stable Micro Systems, Godalming, UK) equipped with a 25 mm diameter cylinder probe. The test settings were as follows: pretest speed—1.0 mm/s; test speed—1.7 mm/s; post-test speed—10.0 mm/s; strain—40%; trigger force—5 g [10]. The bread was compressed twice to provide insight into how samples behave when chewed. The following parameters were recorded by the Exponent software: hardness (the peak force of the first compression), springiness (the distance of the detected height during the second compression divided by the original compression distance), cohesiveness (the area of the second compression divided by the area of the first compression), gumminess (hardness × cohesiveness), chewiness (gumminess × springiness), and resilience (by dividing the upstroke energy of the first compression by the down stroke energy of the first compression).

### 2.7. Color Measurements

Color readings (L*, a*, and b*) of the bread crust and crumb were taken using a tristimulus color analyzer (Minolta Chroma Meter CR 210, Minolta Camera Co., Tokyo, Japan). The illuminant C (CIF, standard, 6774 K) was used. Results were expressed as L* (brightness), a* (redness), and b* (yellowness). The instrument was calibrated using a standard white tile (L* = 98.03, a* = −0.23, b* = 0.25). The change in color due to fish powder addition was determined by calculating the color differential index (∆E) using following equation:

(3) $$∆E\;=\;\sqrt{{\left(∆L\right)}^{2}+{\left(∆a\right)}^{2}+{\left(∆b\right)}^{2}}$$

where ∆L: L*sample − L*control; ∆a: a*sample − a*control; and ∆b: b*sample − b*control.

### 2.8. In Vitro Starch Digestion Process

An in vitro starch digestion process was used to determine the rate and extent of starch digestion of the bread as described in Reference [10]. The process mimicked stomach digestion via the use of 0.8 mL 1M HCl and 1 mL of 10% pepsin (Sigma Aldrich, Saint Louis, MO, USA) per 50 g of bread sample whilst the material was incubated at 37 °C for 30 min under constant stirring. Aliquots (1 mL) were taken (time 0) and added to 4 mL absolute alcohol. Small intestine digestion was mimicked via the addition of enzyme solution (5 mL of 2.5% pancreatin (Sigma Aldrich, Saint Louis, MO, USA) solution in 0.1 M sodium maleate buffer pH 6) with constant stirring at 37 °C for 120 min and aliquots withdrawn after 20, 60, and 120 min and added to 4 mL absolute alcohol. The samples were analyzed for reducing sugar content using 3.5-dinitrosalicylic acid. In all cases, the reducing sugar release of the bread samples was calculated in mg reducing sugar/g sample and plotted against time, and the area under the curve (AUC) was calculated by dividing the graph into trapezoids.

### 2.9. Amino Acid Profile and Scoring

To determine the amino acid profile and value of the samples, the material was hydrolyzed with 6 N hydrochloric acid in an oven at 110 °C for 20 h and the total amount of amino acids in the samples was determined using an Agilent 1100 series (Agilent Technologies, Walbronn, Germany) high-performance liquid chromatography following the methodology proposed in Reference [20]. The extracted amino acid samples were injected into an HPLC equipped with a 150 × 4.6 mm, C18, 3u ACE-111-1546, (Winlab, Glasgow, Scotland) column for amino acid separation. Column flow rate was 0.7 mL/min and the temperature was kept at 40 °C. O-phthaldialdehyde was used as a fluorescence derivative reagent for primary amino acids, and 9-fluorenylmethyl chloroformate for secondary amino acids. Detection utilized a fluorescence detector with an excitation of 335 nm and emission of 440 nm for primary amino acids. At 22 min, the detector was switched to excitation 260 nm, emission 315 nm to detect secondary amino acids such as proline. The amino acid results are expressed in mg of amino acids per g protein. Amino acid scores were calculated by dividing the amino acid content of the bread (mg/g protein) by the suggested reference pattern of amino acid requirements (mg/g protein) for pre-school children (1–2 years old) for nine essential amino acids plus tyrosine and cysteine as follows: histidine—18, isoleucine—31, leucine—63, lysine—52, methionine + cysteine—25, phenylalanine + tyrosine—46, threonine—27, and valine—41 [21].

### 2.10. In Vitro Protein Digestibility, Protein Digestibility Corrected Amino Acid Score (PDCAAS), and Nutritional Index

The multi-enzyme technique described in Reference [22] was used for the determination of in vitro protein digestibility of the bread sample. Protein digestibility of foods is frequently assessed using in vivo rat experimental models as originally proposed in Reference [23]. Aside from in vivo methods, Reference [21] also recommends the use of in vitro methods using enzymes. A 50 mL of protein suspension was prepared in distilled water (6.25 mg of protein/mL), adjusted to pH 8 with a solution of 0.1 N HCL and/or 0.1 N NaOH, and placed on a heated stirring block at 37 °C. The multi-enzyme solution (1.6 mg/mL Trypsin, 3.1 mg/mL chymotrypsin, and 1.3 mg/mL peptidase) was maintained in an ice bath and adjusted to pH 8.0 with 0.1 N HCL and/or 0.1 N NaOH. Five mL of the multi-enzyme solution was then added to the protein suspension, which was maintained at 37 °C. The decrease in pH was measured after the addition of an enzymatic solution at every minute for a period of 10 min using a digital pH meter (S20 Seven EasyTM, Mettler Toledo, Columbus, OH, USA). The percent protein digestibility (Y) was calculated by using Y = 210.46 − 18.10 × X, where X represents the change in pH after 10 min. PDCAAS was calculated by multiplying the IVPD with limiting amino acid score (lowest score of an individual amino acid). The essential amino acid index (EAAI) estimates the quality of the test protein, using its EAA content as the criterion. EAAI was calculated according to the procedure of Oser (1959). It considers the ratio between the EAA of the test protein and the EAA of the reference protein, according to the equation:

(4) $$\sqrt{\frac{\left(EAA1\times 100\right)\left(EAA2\times 100\right)\ldots \ldots \left(EAAn\times 100\right)\left(sample\right)}{\left(EAA1\times 100\right)\left(EAA2\times 100\right)\ldots ..\left(EAAn\times 100\right)\left(reference\right)}}$$

The biological value (BV) indicates the utilizable fraction of the test protein. BV was calculated using the equation of Oser (1959): BV = ((1.09 × EAAI) − 11.70). The nutritional index (NI) normalizes the qualitative and quantitative variations of the test protein compared to its nutritional status. NI was calculated using the equation of Crisan and Sands (1978), which considers all the factors with an equal importance: NI = (EAAI × Protein (%)/100).

### 2.11. Fatty Acid Profile

The composition of the lipids of the bread samples were analyzed using gas chromatography coupled with a flame ionization detector (FID) (Perkin Elmer, Waltham, MA, USA). The fatty acid methyl esters were separated in a VARIAN gas chromatograph model CP 7420 equipped with FID and CP-Sil (100m long, 0.25 mm internal diameter, and 0.2 μm film thickness) fused silica capillary column. An aliquot of the extracted samples (1.0 µL) was injected, and the temperature of the injector (split 1:30) and the detector temperature were 250 °C. The column temperature started at 45 °C with a ramp of 13 °C/min and 4 °C/min until 175 °C and 215 °C which were held for 27 min and 35 min, respectively. Oven temperature was then raised to 250 °C and held for 5 min. The fatty acid content was expressed as a percentage of the total fatty acids detected. The standard solution 68 D was used to establish the correction factors for each of the certified fatty acids, which were used to transform the percentage peaks by weight (mg/g of total fatty acids). The methyl ester was quantified through the integration of the peak area using the software Star 6.0 (Perkin Elmer, Waltham, MA, USA). Helium gas was utilized as the carrier gas with a flow rate of 16.7 cm/s [24].

### 2.12. Determination of Total Phenolic Content

Total phenolic content of supernatant obtained from in vitro gastro-intestinal digestion was measured using the Folin-Ciocalteu method as described in Reference [25]. The phenolic contents were expressed as mg of Gallic acid equivalents (GAE)/g sample.

### 2.13. Analysis of Antioxidant Properties

The DPPH (2,2-diphenyl-1-picrylhydrazyl) assay was used to determine the antioxidant activity of the material using the method as previously described [26]. Trolox (CAS: 53188-07-1, ACROS Organics™, Morris, NJ, USA) was used as a standard to determine the antioxidant activities of the samples and all results were expressed as µmol trolox equivalent (TE) per g sample.

### 2.14. Statistical Analysis

All experiments were performed in triplicate unless otherwise stated. The data were represented as mean ± standard error (SE). Results were subjected to one way analysis of variance (ANOVA) and significance differences were evaluated using Tukey’s comparison test (*p* < 0.05). Statistical software version 16 (Minitab, Melbourne, Australia) was used to perform the statistical analysis of the data.

## 3. Results and Discussion

### 3.1. Chemical Composition and Physical Properties of Bread

Table 1B illustrates the physico-chemical properties of breads that were enriched with different levels of SFP. Inclusion of SFP increased the protein content of the breads from 13.91% in the bread sample without SFP to 20.04% when SFP was added to the bread formula. Similarly, the fat content of SFP-enriched breads were significantly higher (*p* < 0.05) than the control bread. The inclusion of 5–15% SFP to the formulations contributed to a significant decrease (*p* < 0.05) in the carbohydrate content (75–68%) compared to those of the control bread. The lipid content of SFP-enriched breads varied from 6.03 to 9.13% compared to 3.86% in the control bread. The 5–15% SFP supplemented bread exhibited significantly increased lipid content compared to the control bread. The ash contents of SFP breads ranged from 2.36 to 2.42%, which were different (*p* > 0.05) compared to control bread. Samples with added SFP exhibited lower moisture contents as compared to the control breads. Previous research has shown similar results when cobia powder, flaxseed, and lupin have been added to breads [5,13]. The energy value obtained in SFP-enriched breads was higher (*p* < 0.05) than the control bread and this may be related to the higher lipid content of the breads. A similar result has been obtained previously by researchers fortifying bread with cumin and caraway flour [27].

The specific volume of the breads decreased from 2.47 to 2.16 mL/g (Table 1C). Such an observation could be related to the decrease, which was observed in the width/height ratio of breads enriched with SFP when compared to the control bread. It is well known that the gluten components in wheat are responsible for the visco-elastic nature of dough and that this contributes to the shape of the bread by trapping gases during the fermentation stages of bread making. The replacement of wheat flour with SFP would have diluted the gluten content in the doughs and hence contributed to a weaker dough and reduced bread volume. Our findings agree with previous researchers [13] who prepared bread enriched with cobia (*Rachycentron canadum*) flour and found that increasing the cobia flour levels in wheat bread decreased the bread volume. In addition, the different nutrient composition had different effects on the gas production during the fermentation process of bread, thus influencing bread volume [6].

### 3.2. Texture and Color Measurement of Bread

Table 2A illustrates the textural properties of bread. It can be seen that the control bread showed the lowest force required to compress the bread sample (lowest hardness), whereas enriching bread doughs with 5% and 10% SFP caused a significant increase in hardness. The hardness of bread is related to the peak force required to compress the sample, while chewiness represents a quantitative estimation of energy needed to disintegrate bread structure [10]. Hence, it can be postulated that the addition of SFP, and the dilution of the gluten network in the doughs, attributed to the thickening of gas cell walls within the bread crumb and hence the increased level of hardness in the breads. Additionally, the increased levels of protein and lipid that were observed in the SFP-enriched bread samples may be partly responsible for a decrease in gas retention causing unstable gas cells resulting in a more compact structure. Previously, researchers have noted similar observations when combining fish or mussels into bread samples [13,28]. A significant (*p* < 0.05) increase was observed in gumminess and chewiness of SFP-enriched breads compared to the control bread (except at the 15% level). However, the springiness, cohesiveness, and resilience of bread supplemented with SFP were decreased (*p* < 0.05) compared to control bread. Springiness represents the capacity of samples to spring back after a deformation due to the compression. Researchers evaluating the addition of mushroom powders into bread also showed that a reduction in springiness and cohesiveness was related to mushroom powder inclusion and the weakening of the gluten network in the doughs and breads [6].

The lightness (L*), redness (a*), and yellowness (b*) values of the crust and crumbs of bread enriched with SFP are shown in Table 2B. As the levels of SFP increased, the L*, a*, and b* of bread crust decreased. Bread fortified with 20% SFP provided the lowest L*, a*, and b*. For the bread crumbs, the L*, a*, and b* values increased as the level of SFP increased. Bread color is the result of complex reactions that depend on the physico-chemical characteristics of dough (water, starch, and lysine content) and the temperature used during baking process. The addition of SFP alters the crumb color to be more yellowish (b*). The darkening of SFP breads may also be attributed to an increased Maillard reaction taking place during baking due to the higher lysine content. In the Maillard reaction, reducing carbohydrates react with free amino acid side chains of proteins (mainly lysine), and lead to amino acid-sugar reaction products [12]. The results obtained in Reference [29] are similar to the observation of low and high L* and b* values in the bread crust and crumbs, respectively. They reported that the lightness and yellowness values of the bread crust and crumbs increased as the level of amaranth flour (10–40%) powder increased in bread. In addition, it has been shown that the incorporation of lentil flour into bread doughs significantly affected yellowness and lightness characteristics [12]. The ΔE values were determined to evaluate the color differences between the control and the SFP formulations. The ΔE values of the bread crust and crumbs increased (*p* < 0.05) with increasing levels of SFP. In addition, the bread crust exhibited higher ΔE compared to the bread crumbs, which is indicative of the color compounds created as a result of baking. The ΔE values were more than 3.0 for the bread crust and crumbs. According to handbook of color science, these values fall in the “appreciable, detectable by ordinary people”. Reshmi et al. (2017) [30] reported that pomelo enriched bread showed higher ΔE value in bread crust as inclusion increased from 2–7.5%.

### 3.3. Protein Quality of Bread

#### 3.3.1. Amino Acid Profile and Amino Acid Scoring of Bread Samples

Protein quality is considered one of the important characteristics for measuring the nutritional characteristic of a food matrix. SFP inclusion increased (*p* < 0.05) the concentration of essential amino acids (EAA) such as lysine, leucine, isoleucine, methionine, tyrosine, threonine, and valine (Table 3A). Lysine (69–178%), methionine (26–60%), tyrosine (27–42%), and threonine (5–32%) increased in a manner (*p* < 0.05) dependent on the 5–15% SFP inclusion compared to the control bread. However, the levels of isoleucine, leucine, and valine increased more than expected (*p* < 0.05) with 10% and 15% SFP inclusions. The contents of phenylalanine and cysteine decreased in SFP bread compared to the control. No difference was observed in histidine content. Bread prepared with different levels of SFP (SFP15% > SFP10% > SFP5%) exhibited greater (*p* < 0.05) total EAA contents than the control. Reference [31] reported a similar pattern for total EAA levels in bread enriched with faba bean.

The SFP bread had a significantly (*p* < 0.05) higher lysine, isoleucine, leucine, methionine + cysteine, threonine, and valine score (Table 3B). There was no significant difference in phenylalanine + tyrosine and histidine scores compared to the control bread. The scores for isoleucine, leucine, lysine, methionine cysteine, threonine, and valine in SFP bread were higher than the standards for amino acids of an ideal reference protein appropriate for children ages 1–2 (which also covers the range appropriate for human adults) [21]. Based on the essential amino acid scores (Table 3B), the limiting amino acid is lysine for all bread; therefore, the scores for lysine were used to calculate the PDCAAS of the breads. A higher level of SFP addition would, therefore, be required to further improve the lysine score of the bread.

#### 3.3.2. In Vitro Protein Digestibility (IVPD), Protein Digestibility Corrected Amino Acid Score (PDCAAS), and Nutritional Index of Bread

**Figure 3:**
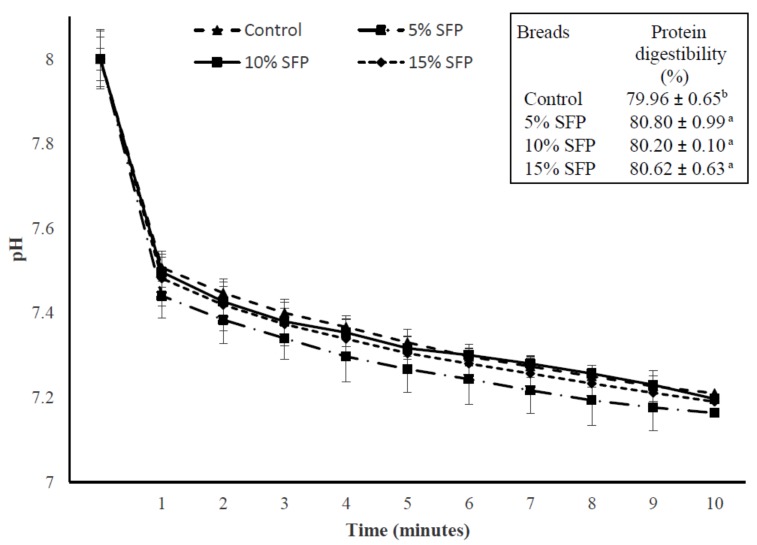
In vitro protein digestibility of bread enriched different levels of salmon fish powder (SFP). Control bread; 5% SFP, 10% SFP, and 15% SFP: bread produced with 5, 10, and 15 g salmon fish powder/100 g wheat flour (*n* = 3 ± standard deviation).

The IVPD and PDCAAS of the SFP bread (Table 3C) ranged from 80.80% to 80.60% and 0.26 to 0.42, respectively. IVPD of SFP bread was higher than the control bread. The pH drop curves of SFP bread using the three enzyme (trypsin, α-chymotrypsin, and protease) system are shown in Figure 3. Previous studies also demonstrated increases in protein digestibility when legume lupin (*Lupinus angustifolius*) flour was added to wheat bread [32]. The addition of SFP to bread significantly affected the IVPD that translated to a significant increase in the PDCAAS. These results indicate that the substitution of wheat flour with SFP at 5–15 g/100 g can potentially increase the IVPD and PDCAAS of wheat bread. This study is the first to report a significant change to IVPD value with the addition of SFP to bread. Compared to the control bread, EAAI and BV were significantly (*p* < 0.05) higher for SFP bread. Among the indices that are used to evaluate the nutritional value of foods, NI combines qualitative and quantitative factors, and it is considered to be a global predictor of the protein quality [33]. Since the protein bioavailability increased, the NI value of SFP breads was significantly (*p* < 0.05) higher than the control bread (Table 3). Similarly, wheat bread enriched with faba bean had significantly (*p* < 0.05) increased the EAAI, BV, and NI indices [31].

### 3.4. Fatty Acid Profile of Bread

SFP bread had lower (*p* < 0.05) SFA and total SFA than the control bread (Table 4). The replacement of SFA using unsaturated fats in the food product reduces the risk of developing cardiovascular diseases [34]. SFP-fortification increased the content of palmitoleic (C16:1), oleic acid (C18:1), and gadoleic acid (C20:1) compared to the control bread and resulted in an increase (*p* < 0.05) of total monounsaturated fatty acid (MUFA) content in SFP bread. The major PUFA present in the control bread was linoleic acid (C18:2). Omega-3 fatty acids were low in content with the ratio of *n*-6:*n*-3 being 12.08:1. This predominance of *n*-6 linoleic acid in cereal is a major contributor to the imbalanced *n*-6:*n*-3 ratio [35]. Increasing the percentage of SFP significantly enhanced EPA and DHA fatty acid content. In turn, the bread prepared with SFP presented an important proportion of this long chain unsaturated fatty acid in relation to other fatty acids. Even in the bread made with 5% SFP, it was possible to obtain an important reduction of the ratio *n*-6:*n*-3 (4.89:1) in comparison with the control bread. From the human nutrition standpoint and prevention of cardiovascular disease, a diet with an *n*-6/*n*-3 ratio between 1 and 5 is recommended by food agencies, scientific societies, and national and international organizations [36]. Similar findings were observed in Reference [34], who reported that the inclusion of chia flour to wheat bread significantly decreased SFA and increased PUFA. Research has indicated that the inclusion of shrimp meat (*Penaeus monodon*) and carp fish powder (*Cyprinus carpio*) could raise this ratio [15,37]. The recommendations of EPA and DHA are 0.250–2 g/day. The consumption of 100 g SFP bread provides 1.07 to 2.06 g of EPA and DHA, which fulfils the minimum daily value recommended. Polyunsaturated fatty acids are easily oxidized in baking processes affecting their stability; however, a significant amount of EPA and DHA remained stable in the bread product. Future studies about the effects of the baking processes of the bread on EPA and DHA content are necessary.

### 3.5. In Vitro Starch Digestion Analysis

**Figure 4:**
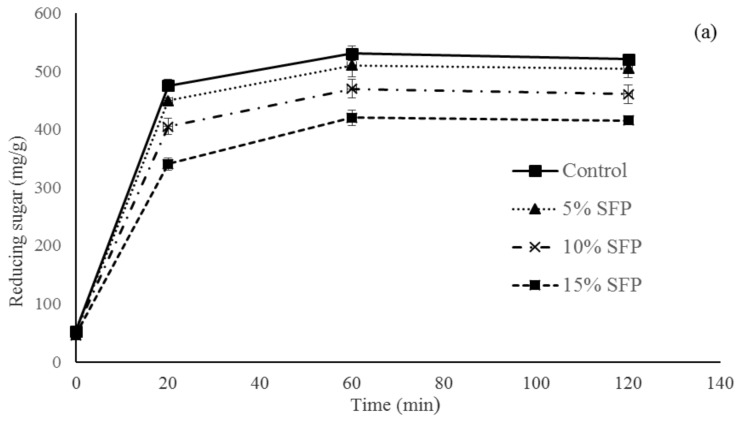

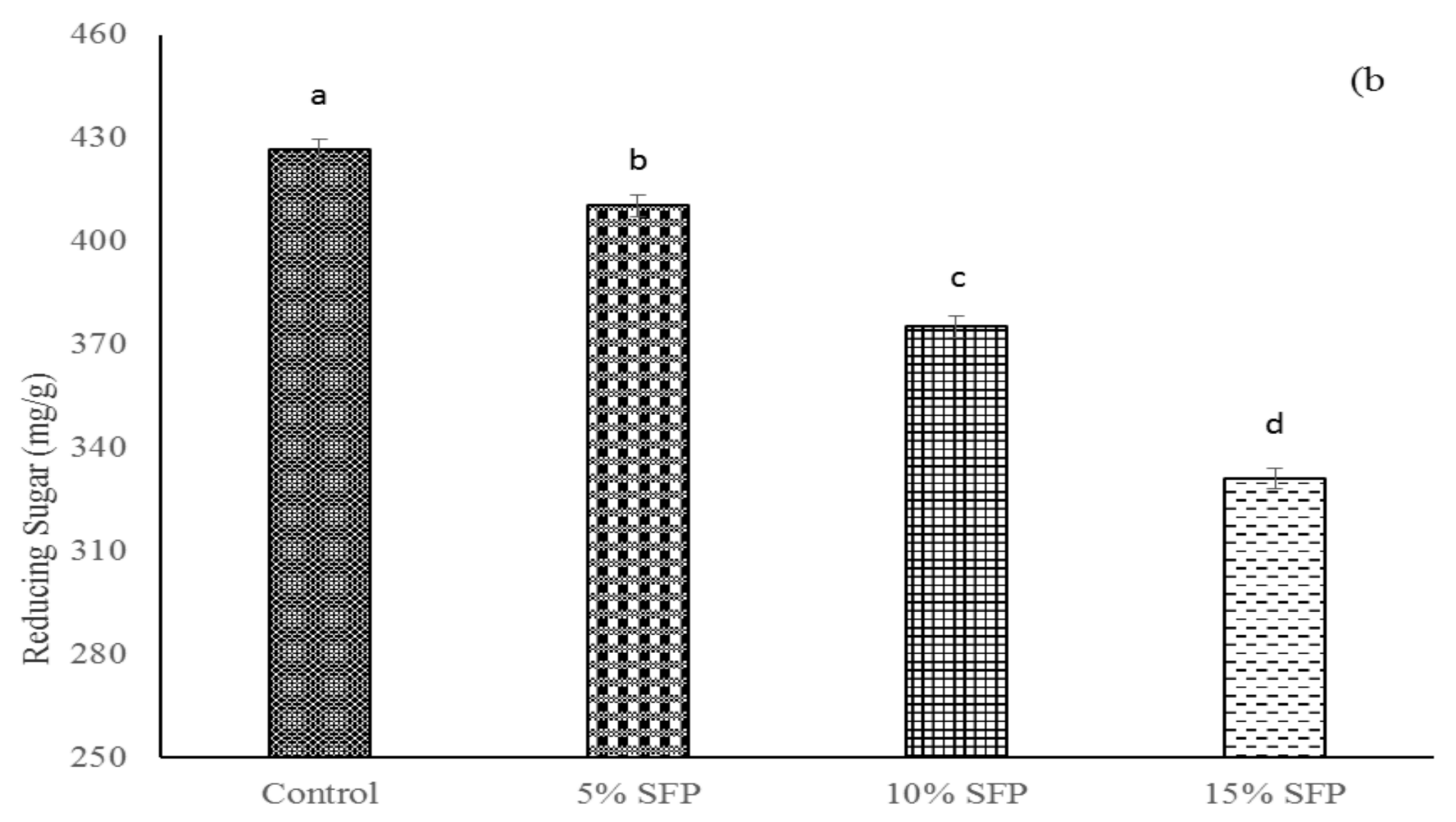
In vitro starch digestibility of bread. (**a**) Amount of reducing sugars released (mg/g starch) during in vitro digestion. (**b**) Values for area under the curve (AUC) compared to the control bread. Control bread; 5% SFP, 10% SFP, and 15% SFP: bread produced with 5, 10, and 15 g salmon fish powder/100 g wheat flour *n* = 3 ± standard deviation. ^a–d^ Values with different superscript letters differ significantly (*p* < 0.05).

Protein, lipid, and starch play an important role in starch digestibility and in the glycemic response in the human body [38,39,40]. It can be seen that there were significantly (*p* < 0.05) more reducing sugars released from the control bread than from the SFP-fortified breads after 20 min of digestion (Figure 4a). The strongest decrease was observed after the addition of 10 and 15 % SFP, followed by 5% SFP bread samples, which could be attributed to higher amount of protein and lipid content in bread fortified with SFP compared to the control bread that formed amylose–lipid complex, which limit the starch availability to starch hydrolyzing enzymes. SFP bread showed significantly higher oleic acid, gadoleic acid, linoleic, and α-linoleic acid compared to the control bread (Table 4). The amylose–lipid interactions results in the formation of single helical structure with a conformational hindrance that restricts enzymes trying to hydrolyze the starch granule. Similar results were reported when investigating the in vitro starch digestibility and glycemic index of millets with different fatty acids palmitic, oleic, and linoleic acids [39]. Additionally, protein rich SFP reduced the starch granule surface accessibility and therefore influenced the enzyme susceptibility. It was previously reported that the presence of protein content in the food matrix creates a strong network and reduces the capacity of enzyme attack to the starch granules, thereby delaying starch digestion [38]. Reshmi et al., 2017 [30] reported that bread fortified with pea flour and pomelo (*Citrus maxima*) fruit segments had a lower glycemic index than control breads. The area under the glucose release curve is a measurement of the glycemic response for 2 h after food is consumed [41]. The values of the areas under the predictive glycemic response curve (AUC) shown in Figure 4b indicate that the addition of SFP to bread significantly decreased the AUC values compared to the control bread. Bread enriched with 5 to 15% SFP had significantly lower AUC values compared to the control bread (Figure 4b). According to previous research, there are several factors that influence the rate of starch digestion, such as type of starch, degree of starch gelatinization, composition, and structure, and non-starch component contents of the starch–protein matrix [6]. Similar results were reported by [6] in which bread fortified with pea flour had a lower glycemic response due to the non-starch components and food matrix effects.

### 3.6. Total Phenolic Content (TPC) and Antioxidant Capacity of Bread

**Figure 5:**
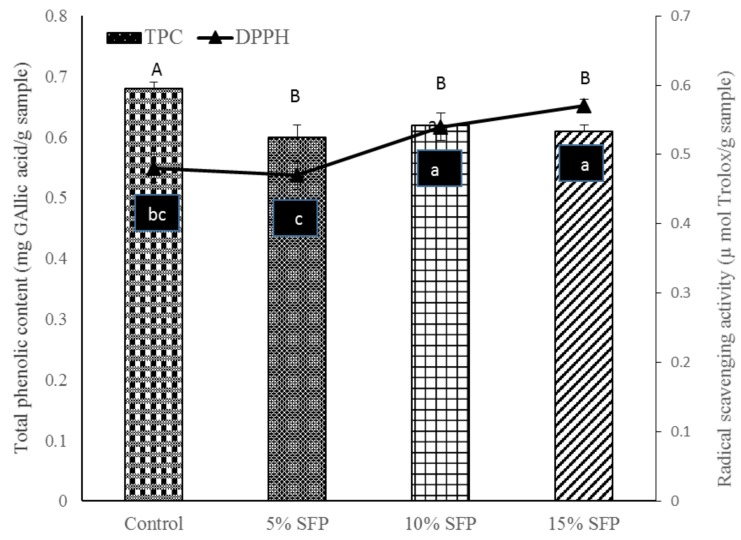
Total phenolic content (mg Gallic acid/g) and radical scavenging activity on the DPPH radical of breads. Control bread; 5% SFP, 10% SFP, and 15% SFP: bread produced with 5, 10, and 15 g salmon fish powder/100 g wheat flour (*n* = 3 ± standard deviation). ^a–c^ Values with different superscript letters differ significantly (*p* < 0.05).

Consumption of food with phenolic-rich ingredients is highly recommended due to their health-promoting effects as they are involved in the prevention of many diseases such as cancers, diabetes, and cardiovascular diseases [27]. SFP bread had lower TPC values than the control bread (Figure 5). This may be due to the thermal deactivation of phenolic compounds during the baking process and formation of indigestible complexes with SFP protein and lipid, such as the enrichment of bread with quinoa leaves, which agrees with previous studies [42]. Phenolic compounds are susceptible to exposure to light, oxygen, and heat, which are normally present in food processing. The total antioxidant activities (TEAC) of 15 % SFP bread were significantly higher at than those of the control bread. Similarly, Reference [43] observed reduced phenolic content and the masking of antioxidant potential of enriched bread with onion skin. Reduction in phenolics could be attributed to phenolics present in the SFP that might have formed protein–phenolics or phenolic–lipid complexes via hydrogen and/or hydrophilic interactions [43].

## 4. Conclusions

Fortification of bread with SFP is an effective technique to improve the protein, essential amino acids, and fatty acids composition of the final product. The addition of SFP significantly increased the protein and energy content of the bread, thus, creating a bread with a higher nutritional value as measured by PDCAAS, BV, and NI, which make them potentially valuable to be used as a source of protein. The results indicate that the inclusion of SFP in bread may give acceptable volume, crumb, and textural properties. SFP bread inhibits starch digestion by increasing the protein matrix around the starch granules, thereby lowering the release of reducing sugars. The antioxidant capacity of the bread was significantly enhanced by SFP inclusion. These results indicate that SFP fortification might be a promising way to produce a product with the maximum potential nutritional and health benefits.

## Figures and Tables

**Table 1:** Bread formulations, proximate compositions, and physical properties of bread. A: Ingredients used in salmon powder (SP) enriched bread. B: Proximate composition of bread elaborated with SP. C: Physical properties of bread made with SP.

**A**							
**Sample code**							
**Ingredients (g)**	**Control**	5% **SFP**	**10% SFP**			**15% SFP**	
Wheat flour	150	142.5	135			127.5	
Salmon powder	-	7.5	15			22	
Salt	2.25	2.25	2.25			2.25	
Sugar	9	9	9			9	
Yeast	2.25	2.25	2.25			2.25	
Butter	7.5	7.5	7.5			7.5	
Water	90	90	90			90	
**B**							
**Sample code**	**SFP**	**Control**	**5% SFP**	**10% SFP**			**15% SFP**
Protein (%)	58.06 ± 0.13	13.91 ± 0.19 ^d^	16.27 ± 0.08 ^c^	18.22 ± 0.06 ^b^			20.04 ± 0.10 ^a^
Fat (%)	38.55 ± 0.09	3.86 ± 0.02 ^d^	6.03 ± 0.06 ^c^	7.26 ± 0.08 ^b^			9.13 ± 0.02 ^a^
Ash (%)	1.37 ± 0.06	2.13 ± 0.02 ^b^	2.36 ± 0.05 ^a^	2.42 ± 0.09 ^a^			2.42 ± 0.09 ^a^
Moisture (%)	2.15 ± 0.03	34.38 ± 0.55 ^a^	31.42 ± 0.42 ^a^	32.90 ± 0.47 ^a^			33.33 ± 0.73 ^a^
Carbohydrate (%)	-	80.10 ± 0.18 ^a^	75.35 ± 0.11 ^b^	72.10 ± 0.19 ^c^			68.42 ± 0.11 ^d^
Energy (Kcal)	579.55 ± 0.30	410.8 ± 0.18 ^d^	420.71 ± 0.17 ^c^	426.62 ± 0.44 ^b^			435.96 ± 0.36 ^a^
**C**							
**Sample code**	**Width/height ratio (mm)**		**Volume (mL)**		**Specific volume (mL/g)**		
Control	1.80 ± 0.12 ^b^		111 ± 1.00 ^a^		2.47± 0.02 ^c^		
5% SFP	1.77 ± 0.38 ^a^		103 ± 1.15 ^b^		2.35 ± 0.01 ^b^		
10% SFP	1.74 ± 0.15 ^a^		97 ± 0.58 ^c^		2.18 ± 0.00 ^a^		
15% SFP	1.77 ± 0.10 ^a^		96 ± 1.05 ^c^		2.16 ± 0.02 ^a^		

5% SFP, 10% SFP, and15% SFP: bread prepared with 5, 10, and 15 g of salmon fish powder/100 g of wheat flour. Control bread. *n* = 3 ± standard deviation. Values within a column followed by the same superscript letter are not significantly different from each other (*p* > 0.05) according to Tukey’s test.

**Table 2:** Technological characteristic of bread enriched with different levels of salmon powder (SB). A: Texture profile analysis. B: Color characteristics of crust and crumb.

**A**				
**Parameter**	**Control**	**5% SFP**	**10% SFP**	**15% SFP**
Hardness (g)	1082.77 ± 0.20 ^b^	1648.48 ± 44.88 ^a^	1525.91 ± 60.81 ^a^	1198.41 ± 78.59 ^b^
Springiness (mm)	0.935 ± 0.03 ^a^	0.892 ± 0.01 ^b^	0.882 ± 0.03 ^b^	0.879 ± 0.01 ^b^
Gumminess (g)	788.99 ± 5.01 ^b^	946.54 ± 5.26 ^a^	962.11 ± 86.19 ^a^	776.15 ± 33.23 ^b^
Chewiness (g)	737.25 ± 10.79 ^b^	835.58 ± 18.70 ^a^	886.89 ± 76.60 ^a^	674.79 ± 42.26 ^c^
Cohesiveness (ratio)	0.73 ± 0.01 ^a^	0.58 ± 0.02 ^b^	0.62 ± 0.02 ^b^	0.62 ± 0.02 ^b^
Resilience (ratio)	0.35 ± 0.01 ^a^	0.27 ± 0.01 ^b^	0.29 ± 0.02 ^b^	0.29 ± 0.01 ^b^
**B**				
**Crust Color**				
**Sample code**	**Control**	**5% SFP**	**10% SFP**	**15% SFP**
L*	91.45 ± 0.32 ^a^	85.46 ± 0.11 ^b^	86.05 ± 0.38 ^b^	85.95 ± 0.22 ^b^
a*	9.97 ± 0.27 ^a^	2.17 ±0.63 ^b^	1.58 ± 0.37 ^bc^	0.66 ± 0.23 ^c^
b*	36.65 ±0.07 ^a^	32.57 ± 0.09 ^b^	33.42 ± 0.91 ^b^	33.73 ± 0.41 ^b^
ΔE	-	10.67 ± 0.34 ^a^	10.49 ± 0.34 ^a^	11.26 ± 1.08 ^a^
**Crumb Color**				
**Sample code**	**Control**	**5% SFP**	**10% SFP**	**15% SFP**
L*	95.31 ± 0.12 ^b^	96.54 ± 0.11 ^a^	96.59 ± 0.16 ^a^	96.31 ± 0.06 ^a^
a*	12.23 ± 0.02 ^c^	12.96 ± 0.23 ^b^	13.23 ± 0.12 ^ab^	13.53 ± 0.12 ^a^
b*	30.92 ± 0.08 ^d^	32.55 ± 0.56 ^c^	33.92 ± 0.26 ^b^	35.54 ± 0.03 ^a^
ΔE	-	2.23 ± 0.69 ^a^	3.41 ± 0.27 ^b^	4.89 ± 0.23 ^c^

5% SFP, 10% SFP, and15% SFP: bread prepared with 5, 10, and 15g of salmon fish powder/100 g of wheat flour. Control bread. *n* = 3 ± standard deviation.Values within a column followed by the same superscript letter are not significantly different from each other (*p* > 0.05) according to Tukey’s test.

**Table 3:** A: Amino acid (AAs) composition (mg/g protein dry weight basis), B: amino acid score and C: nutritional characterization of wheat bread (control bread) and breads enriched with different levels of salmon fish powder.

**A**				
Amino acid	Control	5% SFP	10% SFP	15% SFP
Phenylalanine	42.24 ± 0.19 ^a^	38.12 ± 2.30 ^ab^	40.33 ± 2.50 ^ab^	37.48 ± 0.92 ^b^
Tyrosine	18.66 ± 2.08 ^b^	23.62 ± 1.20 ^ab^	25.81 ± 3.37 ^a^	26.47 ± 0.33 ^a^
Histidine	20.75 ± 1.33 ^a^	17.79 ± 1.12 ^a^	20.42 ± 0.89 ^a^	19.42 ± 0.75 ^a^
Isoleucine	30.31 ± 0.81 ^b^	30.63 ± 1.84 ^b^	34.58 ± 1.87 ^a^	34.64 ± 0.68 ^a^
Leucine	62.18 ± 0.57 ^a^	61.13 ± 3.68 ^a^	68.25 ± 3.72 ^b^	66.50 ± 1.41 ^b^
Lysine	9.69 ± 1.14 ^c^	16.42 ± 1.10 ^b^	23.76 ± 1.68 ^a^	26.94 ± 1.00 ^a^
Methionine	10.88 ± 0.65 ^c^	13.71 ± 0.63 ^b^	16.93 ± 1.19 ^a^	17.42 ± 0.22 ^a^
Cysteine	11.69 ± 0.98 ^a^	10.35 ± 0.57 ^ab^	9.56 ± 0.61 ^b^	8.64 ± 0.52 ^b^
Threonine	23.22 ± 0.22 ^b^	24.46 ± 1.62 ^b^	29.78 ± 1.36 ^a^	30.52 ± 0.59 ^a^
Valine	32.17 ± 0.90 ^b^	32.86 ± 1.93 ^b^	37.50 ± 2.07 ^a^	37.57 ± 0.71 ^a^
**ΣEAAs**	261.75 ± 9.23 ^a^	269.14 ± 8.69 ^b^	306.96 ± 7.89 ^c^	305.65 ± 6.76 ^c^
B. Amino acid score ^a^				
Histidine	1.15 ± 0.08 ^a^	0.99 ± 0.06 ^a^	1.13 ± 0.04 ^a^	1.07 ± 0.04 ^a^
Isoleucine	0.98 ± 0.03 ^b^	0.99 ± 0.05 ^b^	1.12 ± 0.06 ^a^	1.12 ± 0.02 ^a^
Leucine	0.99 ± 0.01 ^a^	0.97 ± 0.05 ^a^	1.08 ± 0.05 ^a^	1.05 ± 0.02 ^a^
Lysine	0.19 ± 0.02 ^c^	0.32 ± 0.02 ^b^	0.46 ± 0.03 ^a^	0.52 ± 0.01 ^a^
Phenylalanine + Tyrosine	1.32 ± 0.05 ^a^	1.34 ± 0.05 ^a^	1.43 ± 0.06 ^a^	1.39 ± 0.02 ^a^
Methionine + Cysteine	0.87 ± 0.06 ^b^	0.92 ± 0.02 ^ab^	1.01 ± 0.01 ^a^	1.00 ± 0.03 ^a^
Threonine	0.86 ± 0.02 ^b^	0.91 ± 0.06 ^b^	1.10 ± 0.05 ^a^	1.13 ± 0.02 ^a^
Valine	0.77 ± 0.03 ^b^	0.78 ± 0.04 ^b^	0.89 ± 0.04 ^a^	0.89 ± 0.01 ^a^
C. Nutritional parameters				
IVPD (%)	79.96 ± 0.65 ^a^	80.80 ± 0.99 ^a^	80.20 ± 0.10 ^a^	80.60 ± 0.73 ^a^
PDCAAS ^b^	0.15 ± 0.06 ^a^	0.26 ± 0.05 ^b^	0.37 ± 0.02 ^c^	0.42 ± 0.06 ^d^
EAAI	62.51 ± 1.15 ^a^	71.44 ± 1.28 ^b^	76.76 ± 1.95 ^c^	76.68 ± 1.40 ^c^
BV	56.44 ± 1.05 ^a^	66.18 ± 1.22 ^b^	71.97 ± 1.56 ^c^	71.68 ± 1.10 ^c^
NI	8.69 ± 0.10 ^a^	11.62 ± 0.19 ^b^	13.98 ± 0.38 ^c^	15.36 ± 0.21 ^d^

Bold values indicate the grand totals for essential amino acids (ΣEAAs). 5% SFP, 10% SFP, and 15% SFP: bread prepared with 5, 10, and 15 g of salmon fish powder/100 g of wheat flour. Control bread. *n* = 3 ± standard deviation. ^a^ Based on standard FAO/WHO/UNU (2007) 1–2-year-old reference pattern (mg/g protein): Histidine—18, Lysine—52, Isoleucine—31, Leucine—63, Methionine + Cysteine—26, Phenylalanine + Tyrosine—46, Therionine—27, Valine—42. ^b^ Protein digestibility corrected amino acid score (PDCAAS): AAS (lowest score of an individual amino acid) × in vitro protein digestibility of bread sample. Values within a column followed by the same superscript letter are not significantly different from each other (*p* > 0.05) according to Tukey’s test. IVPD: in vitro protein digestibility, EAAI: essential amino acid index, BV: biological value, NI: nutritional index.

**Table 4:** Fatty acid profile (g of individual fatty acids/100 g of total fatty acids) of bread enriched with different levels of salmon fish powder (SFP).

Fatty acid	Control	5% SFP	10% SFP	15% SFP
**Saturated Fatty Acids (SFA)**				
C12:0	3.68 ± 0.01 ^a^	2.69 ± 0.02 ^b^	1.99 ± 0.01 ^c^	1.70 ± 0.00 ^d^
C14:0	9.74 ± 0.06 ^a^	7.64 ± 0.07 ^b^	6.11 ± 0.04 ^c^	5.56 ± 0.01 ^d^
C15:0	1.77 ± 0.01 ^a^	1.36 ± 0.01 ^b^	1.08 ± 0.01 ^c^	0.95 ± 0.01 ^d^
C16:0	35.48 ± 1.44 ^a^	31.87 ± 0.08 ^b^	29.14 ± 0.05 ^c^	28.01 ± 0.03 ^d^
C17:0	0.96 ± 0.08 ^a^	0.75 ± 0.08 ^b^	0.62 ± 0.04 ^bc^	0.56 ± 0.04 ^c^
C18:0	8.27 ± 0.11 ^a^	7.25 ± 0.10 ^b^	6.50 ± 0.01 ^c^	6.20 ± 0.01 ^d^
C19:0	0.29 ± 0.01 ^a^	0.20 ± 0.01 ^b^	0.15 ± 0.01 ^b^	0.19 ± 0.01 ^b^
C20:0	0.11 ± 0.01 ^a^	0.12 ± 0.00 ^a^	0.12 ± 0.00 ^a^	0.12 ± 0.00 ^a^
C22:0	-	0.07 ± 0.00 ^c^	0.14± 0.01 ^b^	0.16 ± 0.01 ^a^
C24:0	-	0.06 ± 0.01 ^b^	0.11 ± 0.02 ^a^	0.13 ± 0.01 ^a^
**Monounsaturated Fatty Acids (MUFA)**				
C14:1	0.83 ± 0.01 ^a^	0.63 ± 0.00 ^b^	0.50 ± 0.00 ^b^	0.43 ± 0.00 ^d^
C16:1 ω-7	1.37 ± 0.02 ^d^	2.52 ± 0.01 ^c^	3.31 ± 0.01 ^b^	3.73 ± 0.00 ^a^
C17:1	0.14 ± 0.01 ^b^	0.17 ± 0.00 ^a^	0.18 ± 0.01 ^a^	0.18 ± 0.00 ^a^
C18:1 ω-9	17.28 ± 0.08 ^d^	23.67 ± 0.12 ^c^	28.41 ± 0.01 ^b^	31.09 ± 0.04 ^a^
C20:1	0.03 ± 0.04 ^d^	0.46 ± 0.03 ^c^	0.88 ± 0.02 ^b^	1.05 ± 0.01 ^a^
C22:1 ω-9	-	0.10 ± 0.00 ^c^	0.15 ± 0.01 ^b^	0.17 ± 0.00 ^a^
C24:1 ω-9	-	-	0.07 ± 0.00 ^b^	0.08 ± 0.01 ^a^
**Polyunsaturated fatty acids (PUFA)**				
C18:2 ω-6	12.08 ± 0.09 ^c^	12.68 ± 0.06 ^b^	12.97 ± 0.04 ^a^	12.91 ±0.01 ^c^
C18:3 ω-3	1.35 ± 0.00 ^d^	1.40 ± 0.01 ^c^	1.50 ± 0.01 ^b^	1.45 ± 0.00 ^a^
C20:2 ω-6	-	0.10 ± 0.01 ^c^	0.15 ± 0.00 ^b^	0.17 ± 0.00 ^a^
C20:3 ω-6	-	0.09 ± 0.01 ^c^	0.14 ± 0.00 ^b^	0.17 ± 0.00 ^a^
C20:4 ω-6	-	0.14 ± 0.01 ^c^	0.22 ± 0.01 ^b^	0.25 ± 0.00 ^a^
C20:5 ω-3	-	0.29 ± 0.00 ^c^	0.46 ± 0.00 ^b^	0.58 ± 0.01 ^a^
C22:2 ω-6	-	-	-	0.04 ± 0.01
C22:5 ω-3	0.09 ± 0.04 ^d^	0.18 ± 0.01 ^c^	0.25 ± 0.00 ^b^	0.28 ± 0.00 ^a^
C22:6 ω-3	-	0.78 ± 0.03 ^c^	1.31 ± 0.01 ^b^	1.54 ± 0.01 ^a^
ΣSFA	60.31 ± 0.45 ^a^	49.33 ± 0.41 ^b^	43.96 ± 0.27 ^c^	43.60± 0.12 ^c^
ΣMUFA	19.65 ± 0.17	27.56 ± 0.18	33.50 ± 0.12	36.75 ± 0.06
ΣPUFA	13.51 ± 0.14	15.68 ± 0.13	17.00 ± 0.08	16.62 ± 0.04
Σω-6	12.08 ± 0.01	13.01 ±0.02	13.48 ± 0.05	12.83 ± 0.01
Σω-3	1.43 ± 0.01	2.66 ± 0.04	3.52 ± 0.03	3.80 ± 0.02
PUFA/SFA	0.22	0.31	0.38	0.38
EPA + DHA	-	1.07 ± 0.03	1.77 ± 0.01	2.06 ± 0.01
ω-6/ω-3	12.08	4.89	3.82	3.37

5% SFP, 10% SFP, and 15% SFP: Bread prepared with 5, 10, and 15 g of salmon fish powder/100 g of wheat flour. Control bread sample. *n* = 3 ± standard deviation. Values within a column followed by the same superscript letter are not significantly different from each other (*p* > 0.05) according to Tukey’s test.
